# FHND004 inhibits malignant proliferation of multiple myeloma by targeting PDZ-binding kinase in MAPK pathway

**DOI:** 10.18632/aging.205634

**Published:** 2024-03-07

**Authors:** Hongjie Wu, Jinjun Qian, Lianxin Zhou, Tingting Hu, Yuanjiao Zhang, Chen Wang, Ye Yang, Chunyan Gu

**Affiliations:** 1 Nanjing Hospital of Chinese Medicine Affiliated to Nanjing University of Chinese Medicine, Nanjing, China; 2 School of Medicine and Holistic Integrative Medicine, Nanjing University of Chinese Medicine, Nanjing, China

**Keywords:** multiple myeloma, PDZ-binding kinase, MAPK signaling, FHND004, proliferation

## Abstract

Inhibitors of Epidermal growth factor receptor tyrosine kinase (EGFR-TKIs) are producing impressive benefits to responsive types of cancers but challenged with drug resistances. FHND drugs are newly modified small molecule inhibitors based on the third-generation EGFR-TKI AZD9291 (Osimertinib) that are mainly for targeting the mutant-selective EGFR, particularly for the non-small cell lung cancer (NSCLC). Successful applications of EGFR-TKIs to other cancers are less certain, thus the present pre-clinical study aims to explore the anticancer effect and downstream targets of FHND in multiple myeloma (MM), which is an incurable hematological malignancy and reported to be insensitive to first/second generation EGFR-TKIs (Gefitinib/Afatinib). Cell-based assays revealed that FHND004 and FHND008 significantly inhibited MM cell proliferation and promoted apoptosis. The RNA-seq identified the involvement of the MAPK signaling pathway. The protein chip screened PDZ-binding kinase (PBK) as a potential drug target. The interaction between PBK and FHND004 was verified by molecular docking and microscale thermophoresis (MST) assay with site mutation (N124/D125). Moreover, the public clinical datasets showed high expression of PBK was associated with poor clinical outcomes. PBK overexpression evidently promoted the proliferation of two MM cell lines, whereas the FHND004 treatment significantly inhibited survival of 5TMM3VT cell-derived model mice and growth of patient-derived xenograft (PDX) tumors. The mechanistic study showed that FHND004 downregulated PBK expression, thus mediating ERK1/2 phosphorylation in the MAPK pathway. Our study not only demonstrates PBK as a promising novel target of FHND004 to inhibit MM cell proliferation, but also expands the EGFR kinase-independent direction for developing anti-myeloma therapy.

## INTRODUCTION

Multiple myeloma (MM) is a malignancy of plasma cells in the bone marrow, and mainly occurs among older adults (median age around 70) with clinical manifestations such as hypercalcemia, anemia, renal insufficiency, and pathological fractures [1–3]. In the last two decades, the use of novel drugs such as proteasome inhibitors (PI), immunomodulatory drugs, antibody-based targeting immunotherapy, and chimeric antigen receptor (CAR) T-cell therapy have improved the overall survival rate of MM patients. However, it remains incurable as most patients unavoidably relapsed and became drug resistance [4–6]. Thus, there is an urgent need to find more potential drugs/targets and develop more therapeutic strategies.

Since the approval of the first designed kinase inhibitor (imatinib) in 2001 and the first anti-EGFR inhibitor (Gefitinib) in 2003 [7, 8], great efforts have been devoted to the rational design and improvement of new kinase inhibitors. There are two main types of EGFR inhibitors: monoclonal antibodies (mAbs, such as Cetuximab) and small molecule tyrosine kinase inhibitors (TKIs, including Gefitinib and Afatinib as the 1st and 2nd generation) [9]. These EGFR-TKIs are mainly used to treat NSCLC harboring EGFR exon-19 deletions and the exon-21 L858R mutation [10–12]. However, most patients developed severe TKI drug resistance within 1-2 years mainly due to the additional acquired T798M mutation in EGFR. Third-generation EGFR-TKI AZD9291 (Osimertinib) was highly influential in EGFR-T798M mutated NSCLC, but the adverse effects especially cardiotoxicity are severe [13, 14]. Despite impressive effect on NSCLC patients, EGFR-TKI therapies have limited efficacies and have been clinically challenged by the intrinsic and acquired resistance in the clinic [15, 16]. In order to reduce its cardiotoxicity and overcome the drug resistance caused by T790M/L858R double mutations meanwhile keeping good anti-tumor activity *in vivo* and *ex vivo*, a series of 5,6-dihydro-4H-pyrrolo[3,2,1-ij]quinoline derivatives (namely FHND serial new drugs pipeline from Jiangsu Chia Tai Fenghai Pharmaceutical Co., Ltd., including FHND004 and FHND008) were synthesized and obtained on the basis of AZD9291 structure via modifying the pyrimidine ring or expanding an indole ring [17, 18]. Additionally, considering the complicate intra- and inter-tumor heterogeneity, the successful applications of EGFR-TKIs to other cancers are less certain, largely because these kinase-activating mutations and T790M frequently occur in NSCLC and glioblastoma, but are rarely found in other types of cancers [19, 20]. Though it was reported that first/second generation EGFR-TKIs (Gefitinib/Afatinib) exhibit moderate or no inhibition in NRAS wildtype or mutant MM cells (LP-1 and L-363, respectively) within the EGFR signaling pathway [21], inadvertently, we found that the FHND004 from modifying the third-generation EGFR-TKI AZD9291 (Osimertinib) might have a satisfactory antitumor effect in MM cells (ARP1 and H929). Therefore, this study aims to further explore the FHND drug’s anti-myeloma effect, downstream targets and underlying mechanisms.

In the present study, we verified that FHND004 could inhibit MM proliferation both *in vitro* and *in vivo*. Its potential downstream target, PBK (PDZ-binding kinase, also known as T-lymphokine-activated killer cell-originated protein kinase, TOPK), was distinguished by proteomic chip and RNA-seq methods. The mechanistic studies indicated that FHND004 could inhibit ERK1/2 phosphorylation (p-ERK1/2) by targeting PBK. These findings suggested that using FHND004 to target PBK might be a promising treatment strategy for MM.

## RESULTS

### FHND004 and FHND008 inhibit cellular proliferation in MM cell lines

**Figure 1:**
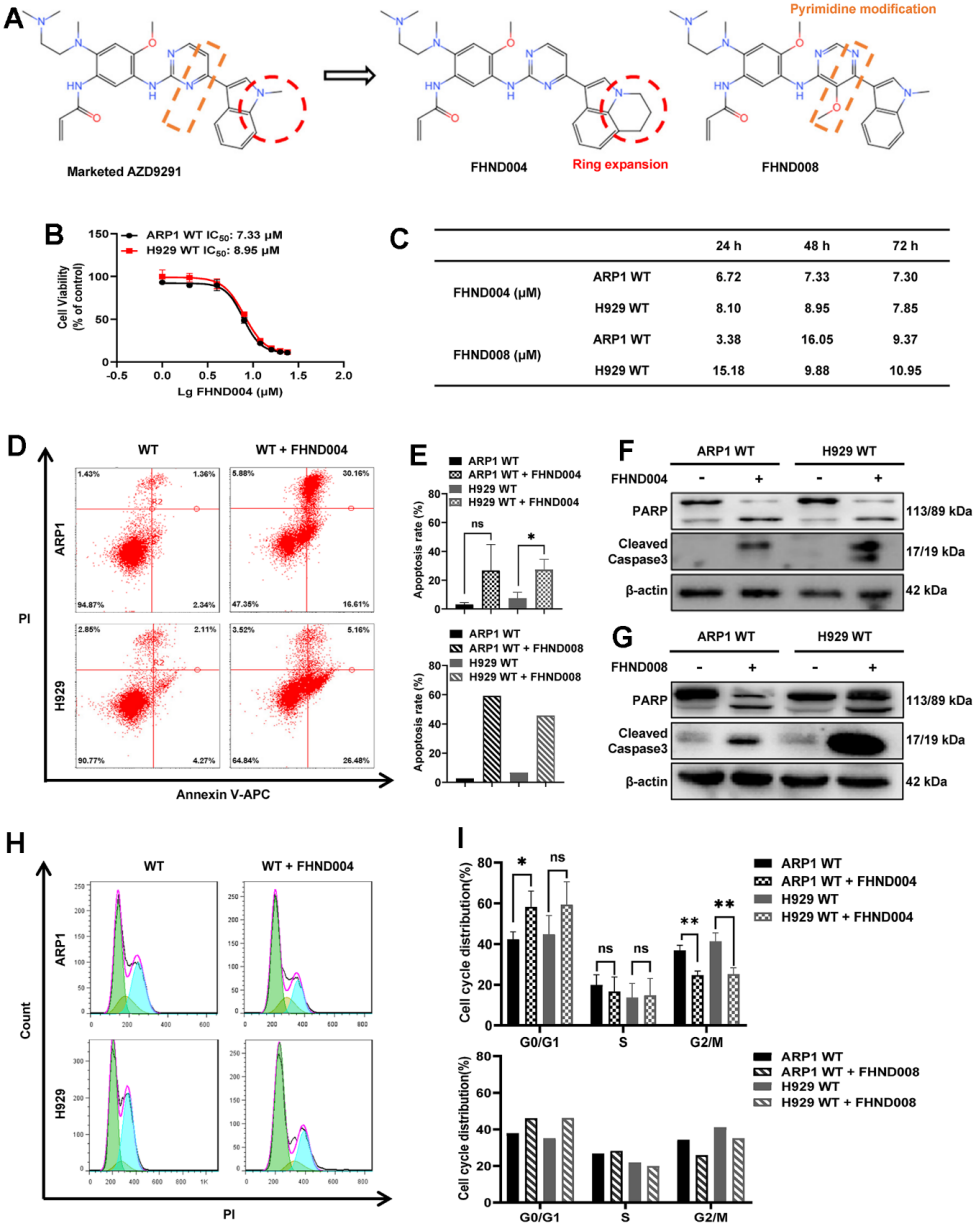
**FHND004 and FHND008 inhibit cellular proliferation in MM cell lines.** (**A**) The chemical structure of FHND004 and FHND008 were modified from the marked third-generation EGFR-TKI AZD2921. (**B**, **C**) Effects of FHND004 and FHND008 on viability of ARP1 WT and H929 WT cells. (**D**) Effects of 24 h treatment with FHND004 (4 μM) on cell apoptosis of ARP1 WT and H929 WT cells were determined by flow cytometry. (**E**) The apoptotic rate of ARP1 WT and H929 WT cells after 24 h treatment with FHND004 (4 μM) and FHND008 (4 μM) was determined quantitatively as histograms. (**F**, **G**) The expressions of PARP, cleaved caspase-3 and β-actin were detected by WB analysis after 24 h treatment with FHND004 (4 μM) and FHND008 (4 μM). (**H**) Effects of 48 h treatment with FHND004 (4 μM) on the cell cycle phases distribution of ARP1 WT and H929 WT cells was determined by flow cytometry. (**I**) The distributions of different cell cycle phases of ARP1 WT and H929 WT cells after 48 h treatment with FHND004 (4 μM) and FHND008 (4 μM) were determined quantitatively. The data of FHND004 were expressed as the mean ± SD; *p* < 0.05 (*), *p* < 0.01 (* *) and *p* < 0.001 (* * *), n = 3.

To determine the antitumor activity of FHND drugs in MM, the MTT assays were performed in both ARP1 and H929 cells for a series of FHND drugs (Supplementary Table 1). Among them, FHND004 and FHND008, based on the marketed AZD9291 (Osimertinib) structure with an expanded indole ring or a modified pyrimidine ring, respectively (Figure 1A), had a relatively better inhibitory activity on the cell viability of MM cells. FHND004 had an impressive anti-proliferation activity in these two MM cells within three days, while FHND008 had similar trends but with higher IC_50_ values (Figure 1B, 1C). Furthermore, the Annexin V/PI staining assay showed that FHND004 and FHND008 could apparently trigger a higher level of apoptosis in these two MM cells at the dosage of 4 μM (Figure 1D, 1E). Western Blotting (WB) results confirmed the up-regulated expression of apoptosis markers: the cleaved PARP (poly ADP-ribose polymerase) and the cleaved caspase-3 after FHND004 and FHND008 treatments (Figure 1F, 1G and quantified in Supplementary Figure 1A, 1B). In addition, the flow cytometry of cell cycle results demonstrated that treatment using FHND004 and FHND008 increased the proportion of cells in the G0/G1 phase and decreased the proportion of cells in the G2/M phase (Figure 1H, 1I). Collectively, these results indicate that FHND004 and FHND008 possess inhibitory activity on MM cell proliferation.

### FHND004 and FHND008 decrease the proliferation of MM cells through the MAPK pathway

**Figure 2:**
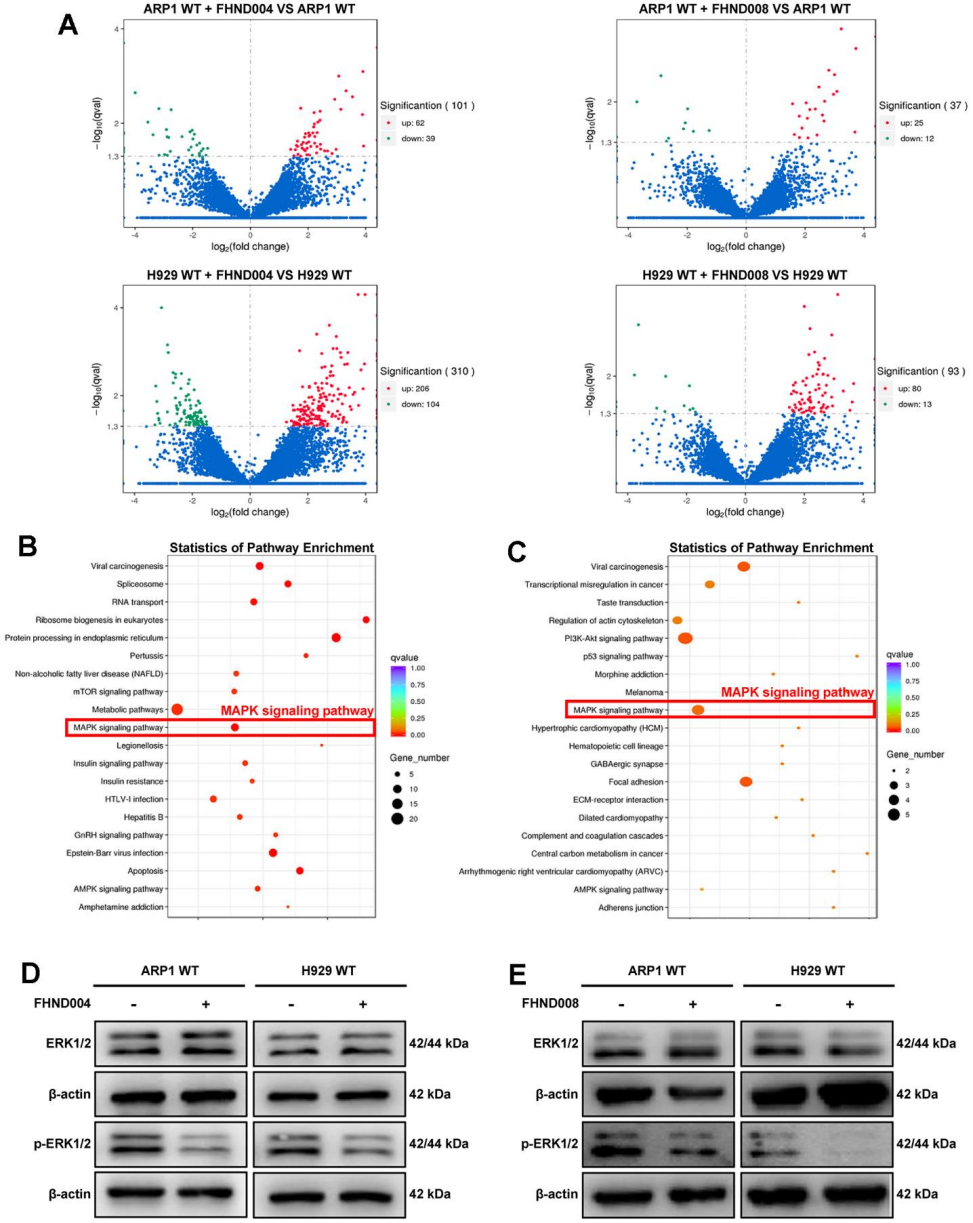
**FHND004 and FHND008 decrease the proliferation of MM cells through the MAPK pathway.** (**A**) The volcano plot of gene expression in ARP1 WT and H929 WT with or without the treatment of FHND004 and FHND008, the red dots indicating significantly upregulated genes and green dots indicating significantly downregulated genes, and blue dots indicating those genes with no significant differential expressions. (**B**, **C**) KEGG pathway analysis of the RNA-seq data indicated that FHND004 and FHND008 were associated with the MAPK signaling pathway. (**D**, **E**) WB analysis of ERK1/2 and p-ERK1/2 expression in ARP1 WT, H929 WT with or without treatment of FHND004 (4 μM) (**D**) and FHND008 (4 μM) (**E**).

To further investigate the potential targets and the downstream signaling pathways of FHND004 and FHND008, we performed transcriptomic RNA-seq in these two MM cell lines with or without FHND004/FHND008 treatment. The volcano plots showed the tens of or hundreds of differentially expressed genes (DEGs) upon FHND004 or FHND008 treatment in ARP1 WT and H929 WT cells, respectively (Figure 2A). The KEGG analysis for these DEGs showed that the MAPK signaling was significantly upregulated in MM cells after the treatment of FHND004 or FHND008 (Figure 2B, 2C). Therefore, we next verified their effect on the expression of key MAPK markers, namely ERK1/2 and its phosphorylated form (p-ERK1/2). The WB results confirmed that the expression profile of p-ERK1/2 protein level but not the total ERK1/2 was significantly reduced by the addition of FHND004 (Figure 2D and quantified in Supplementary Figure 1C) in ARP1 WT and H929 WT cells, and similar results were obtained after the treatment of FHND008 (Figure 2E and quantified in Supplementary Figure 1D). Therefore, it is suggested that FHND004 and FHND008 could suppress MM cells proliferation via the MAPK pathway.

### PBK is identified as a novel target of FHND004 and FHND008

**Figure 3:**
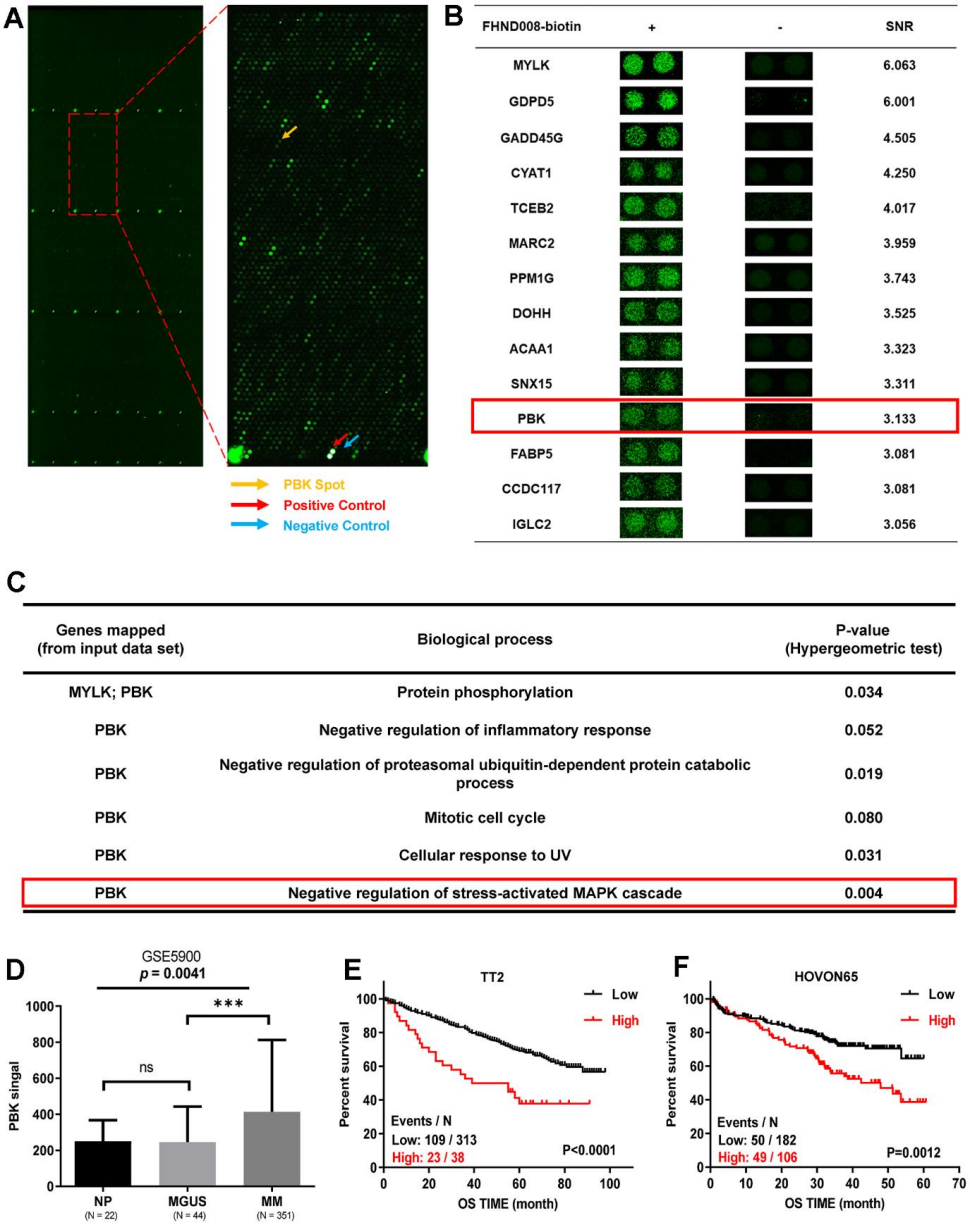
**PBK is identified as a novel target of FHND004 and FHND008.** (**A**) The protein chip scan. The red arrow pointed to the positive control, the blue arrow pointed to the negative control, yellow arrow indicated the PBK protein. (**B**) Top 14 potential FHND008-associated proteins, including PBK. SNR: signal-to-noise ratio. (**C**) Biological processes of PBK protein based on the Gene ontology annotation, PBK is related to the negative regulation of MAPK. (**D**) The mRNA levels of PBK were significantly increased in MM samples. The signal level of PBK was shown on the y-axis. Patients designated as healthy donors with normal bone marrow plasma cells (NP, n = 22), monoclonal gammopathy of undetermined significance (MGUS, n = 44), or multiple myeloma (MM, n = 351) were sorted on the x-axis. (**E**, **F**) Kaplan-Meier analysis revealed the association of PBK expression with overall survival (OS) in TT2 (**E**) and HOVON65 (**F**) cohorts by log-rank test. Events/N means events of death/total patients. The data were expressed as the mean ± SD; *p* < 0.05 (*), *p* < 0.01 (* *) and *p* < 0.001 (* * *).

To explore the direct targets of FHND004 and FHND008, we employed HuProt™ human proteomic chip V4.0 containing 21000 human proteins with 81% coverage and 89% full-length genes, which is suitable for the global high-throughput screen of protein-protein interaction and targets of small molecules [22–24]. According to the experimental standards, the protein chip analysis using biotinylated FHND drugs with positive and negative controls screened out the top 14 protein candidates (Figure 3A, 3B, and Supplementary Table 2). Among them, we next focused on PBK because only the biological process of PBK is related to the negative regulation of MAPK (Figure 3C). At the same time, the GEP analysis of public clinical datasets (GSE2658, GSE5900) for MM cohorts demonstrated that PBK mRNA was increasingly expressed from the “premalignant” plasma cells with monoclonal gammopathy of undetermined significance (MGUS, n = 44) to plasma cells of MM patients (MM, n = 351), compared with normal plasma cells (NP, n = 22) during MM progression (Figure 3D). Additionally, the Kaplan-Meier analysis indicated that the higher PBK expression favored the poorer clinical outcomes for MM patients from the cohorts of TT2 (total therapy 2 from University of Arkansas, GSE2658) as well as HOVON65 (hematology-oncology group-65 from the Dutch-Belgian cooperative trial group, GSE19784) (Figure 3E, 3F). Thus, we speculate that PBK may be a novel direct target of FHND004 and FHND008.

### The affinity of FHND004 to PBK protein is better than FHND008

**Figure 4:**
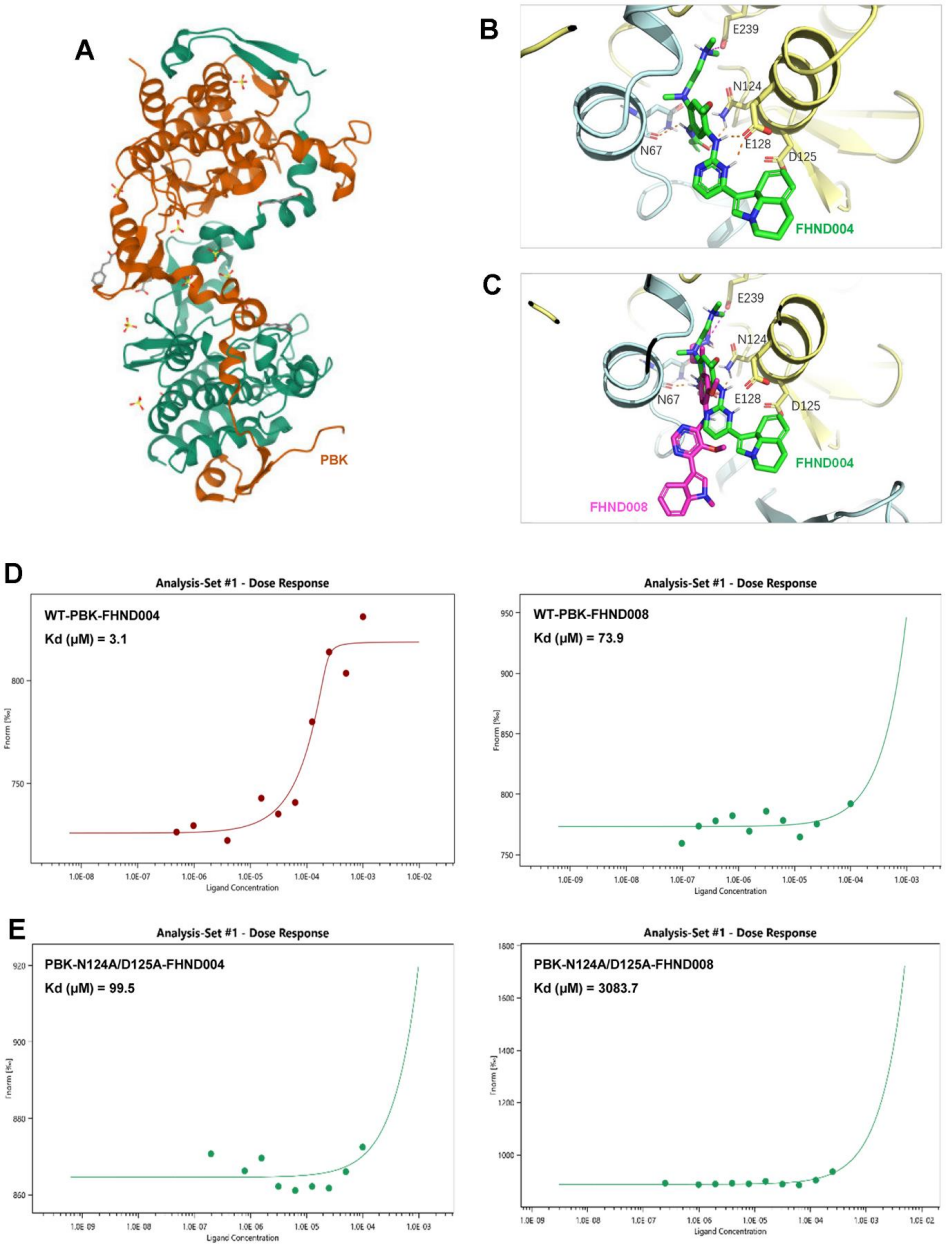
**The affinity of FHND004 to PBK protein is better than FHND008.** (**A**) The structure of PBK (PDB: 5J0A). (**B**, **C**) The molecular docking of FHND004 and FHND008 with PBK. The green stick in B is FHND004, the red stick in C is FHND008, the PBK is represented by a yellow cartoon, and the hydrogen bond is represented by a dotted line. (**D**) MST results of FHND004 and FHND008 on PBK wild type. The results showed that the affinity between FHND004 and PBK was better than that of FHND008. (**E**) MST results after double mutation of N124A and D125A in PBK. The results showed that the affinity of PBK double mutation to FHND004 and FHND008 was significantly decreased.

To further confirm the interactions between PBK and FHND004/FHND008, we then performed molecular docking of FHND004 and FHND008 with the PBK complex (PDB: 5J0A). The result showed that FHND004 and FHND008 could be docked into PBK via interaction with several critical residues, such as N124 and D125 (Figure 4A–4C). Consistently, the MST results showed that the binding affinity of FHND004 with PBK (3.1 μM) was apparently better than that of FHND008 (73.9 μM) (Figure 4D). Meanwhile, double mutation of PBK at sites of N124 and D125 from asparagine and aspartic acid to alanine greatly reduced the affinity of FHND004 (99.5 μM) and FHND008 (3083.7 μM) by 32-fold and 41.7-fold, respectively (Figure 4E). These results support that PBK is the direct target of FHND004 and FHND008 *in vitro*.

### FHND004 exerts anti-tumor activity in CDX and PDX mouse models

**Figure 5:**
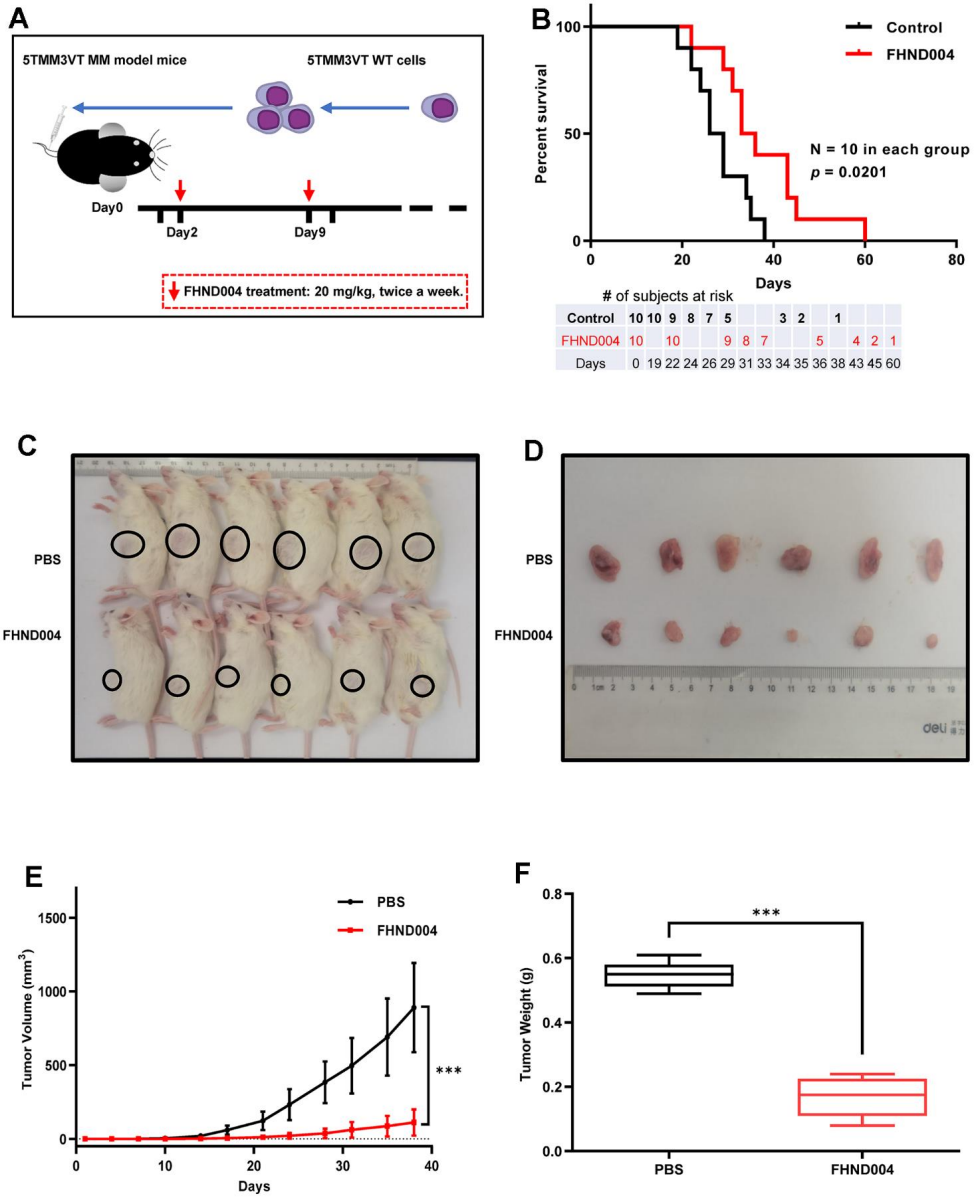
**FHND004 exerts anti-tumor activity in CDX and PDX mouse models.** (**A**, **B**) FHND004 treatment improved the survival of MM-prone C57BL/KaLwRij mice (n = 10, i.g. 20 mg/kg, twice a week). The table at the bottom of the survival curve showed the number of subjects at risk at each time point. (**C**, **D**) Tumor images of PDX mice in the control group (PBS) and the FHND004-treated group (n = 6, FHND004: i.g. 20 mg/kg, twice a week). (**E**) The time course of tumor growth in the control group (PBS) and the FHND004 treated group. (**F**) Mean tumor weights of the control group (PBS) and the FHND004 treated group. The data were expressed as the mean ± SD; *p* < 0.05 (*), *p* < 0.01 (* *) and *p* < 0.001 (* * *).

To verify whether FHND004 could inhibit MM progress *in vivo*, we followed to construct a 5TMM3VT cell-derived xenograft (CDX) MM mouse model at first (Figure 5A). The FHND004 treatment (20 mg/kg body weight, twice per a week) delayed the onset of hind limb weakness and significantly prolonged the survival of myeloma mice than control group (*p* < 0.05) (Figure 5B). In addition, we also verified the anticancer effect of FHND004 on MM cell proliferation by constructing a patient-derived xenograft (PDX) mouse model. The administration of FHND004 obviously inhibited the tumor size compared with the PBS-treated control group (Figure 5C, 5D). Consistently, the smaller average volume and mean weight of tumor mass from the FHND004 group were statistically significant compared to the PBS-control group (*p* < 0.05) (Figure 5E, 5F). Collectively, these data reveal that FHND004 exerts anti-tumor activity *in vivo*.

### FHND004 targets PBK to inhibit MM cell proliferation

**Figure 6:**
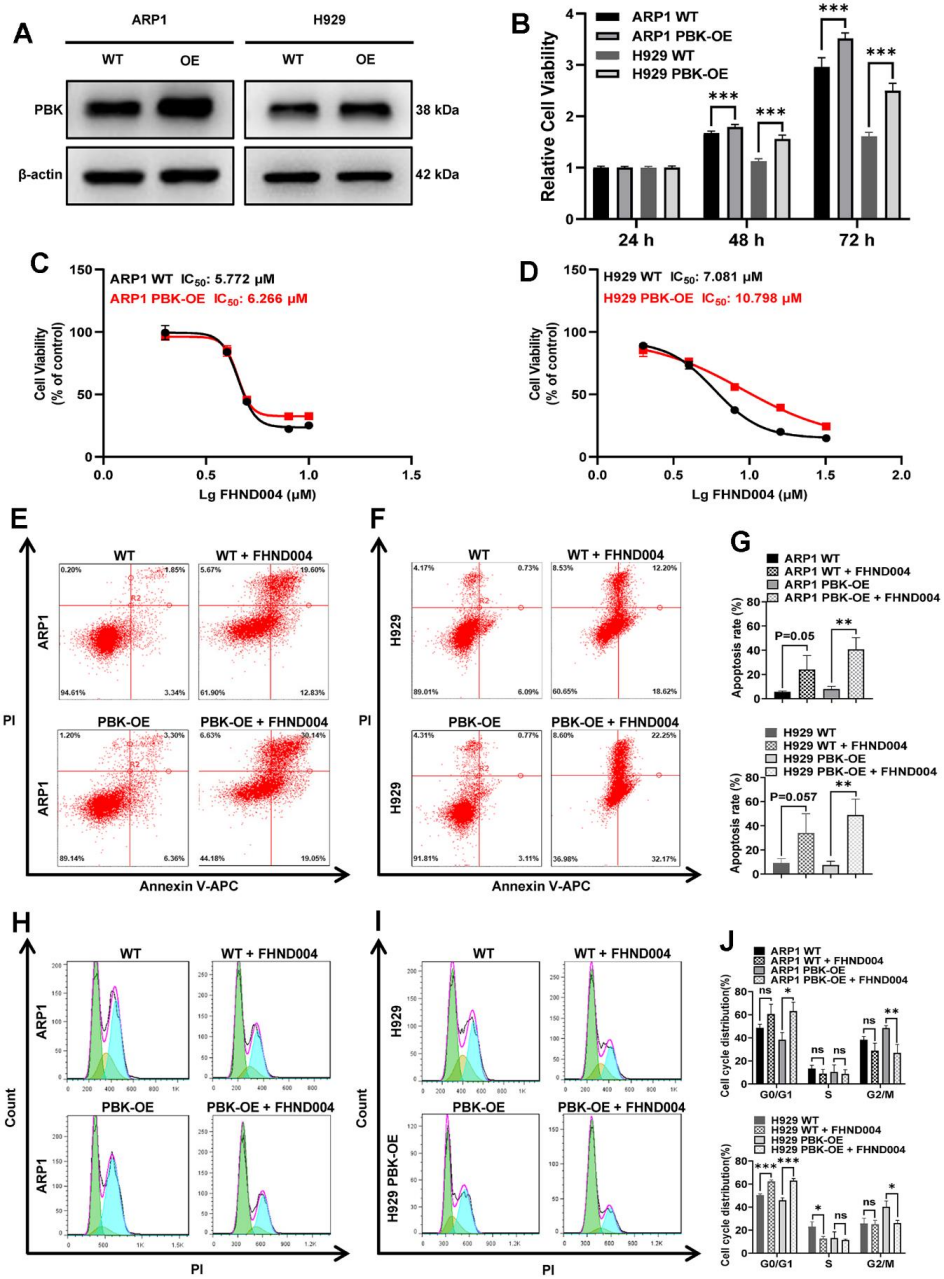
**FHND004 targets PBK to inhibit MM cell proliferation.** (**A**) WB analysis confirmed that PBK-OE MM cells were constructed successfully verified. (**B**) The proliferation of PBK-OE and PBK-WT cells was detected by CCK-8. (**C**, **D**) Effects of 48 h treatment with FHND004 on the viability of ARP1 (**C**) and H929 (**D**) WT and PBK-OE cells. (**E**–**G**) Effects of 24 h treatment with FHND004 (4 μM) on the apoptosis of ARP1 (**E**) and H929 (**F**) WT and PBK-OE cells. (**H**–**J**) Effects of 48 h treatment with FHND004 (4 μM) on the cell cycle phases of ARP1 (**H**) and H929 (**I**) WT and PBK-OE cells. The data were expressed as the mean ± SD, n = 3; *p* < 0.05 (*), *p* < 0.01 (* *) and *p* < 0.001 (* * *).

To further examine the inhibitory effect of drug FHND004 on the proliferation of MM cells through targeting PBK, we constructed ARP1 and H929 cells with stable PBK overexpressing (PBK-OE), which was verified by WB (Figure 6A and quantified in Supplementary Figure 2A). The cell viability assay of CCK-8 results showed that PBK-OE cells had a higher proliferation rate compared to control wild type (WT) cells (Figure 6B). Additionally, PBK overexpression seemed to cause MM cells more insensitive to FHND004, as evidenced by the IC_50_/μM value (in ARP1 WT: 5.772 vs PBK-OE: 6.226; H929 WT: 7.081 vs PBK-OE: 10.798) (Figure 6C, 6D). Furthermore, the apoptosis rate of the PBK-OE treated group was significantly higher than that in PBK-WT treated group, indicating that FHND004 targeting PBK could promote the apoptosis of MM cells (Figure 6E–6G). Consistent with previous results, the cell cycle analysis showed that overexpression of PBK significantly enhanced the proportion of cells in the G2/M phase, while FHND004 significantly decreased the proportion of the G2/M phased cells and increased the proportion of the G0/G1 phased cells, especially for H929 cells (Figure 6H–6J). Therefore, it is indicated that FHND004 inhibits MM cell proliferation via targeting PBK.

### FHND004 targets PBK leading to the inhibition of ERK1/2 phosphorylation in the MAPK pathway

**Figure 7:**
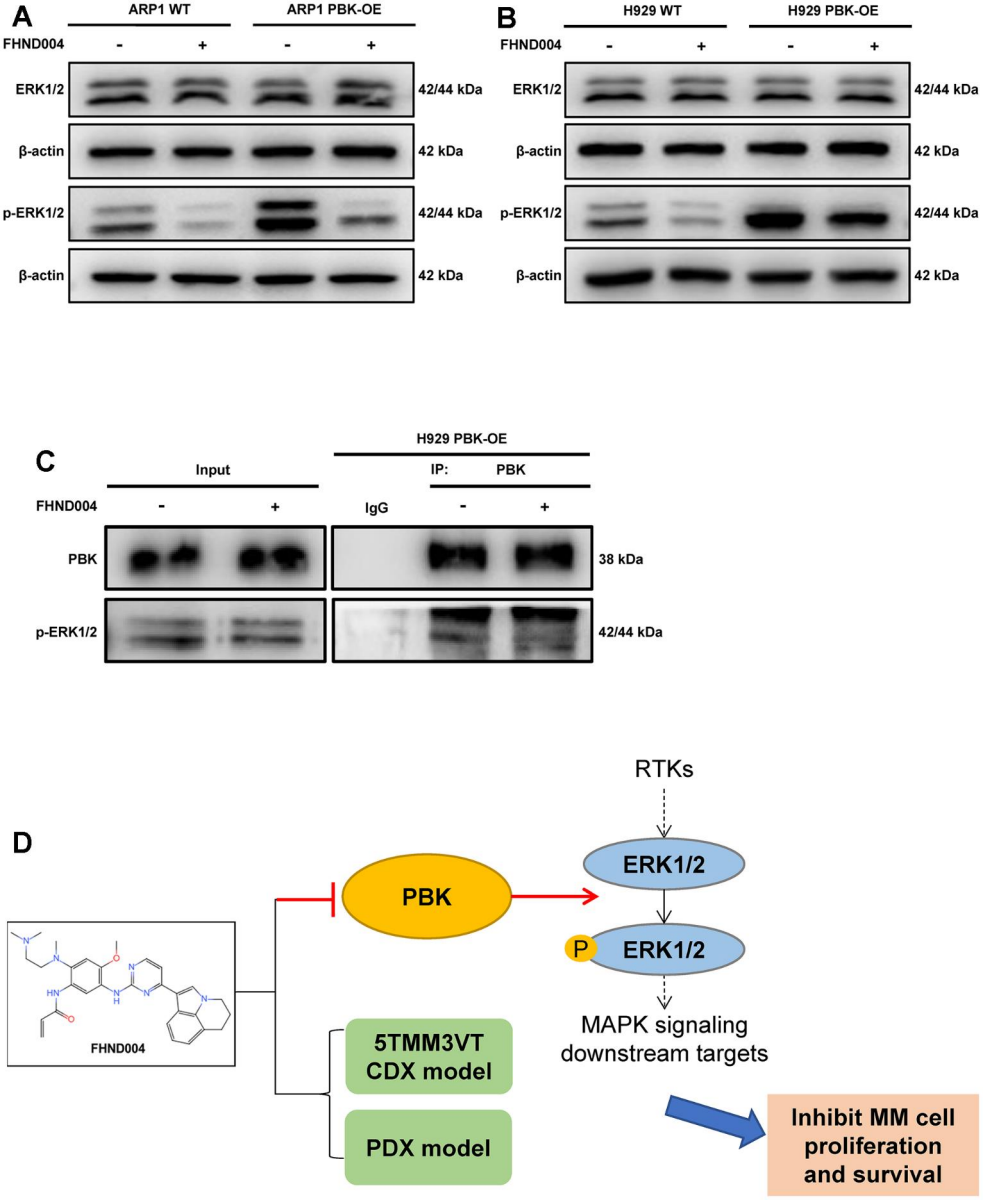
**FHND004 targets PBK leading to the inhibition of ERK1/2 phosphorylation in the MAPK pathway.** (**A**, **B**) WB validated the protein expression profiles of ERK1/2 and p-ERK1/2 in PBK-WT and PBK-OE cells with or without treatment of FHND004 (4 μM) (**A**: ARP1, **B**: H929). n = 3. (**C**) Co-IP assay revealed that FHND004 (4 μM) interfered with the interaction between PBK and p-ERK1/2 in H929 PBK-OE cells. (**D**) Schematic diagram of the mechanism of FHND004 targeting PBK in MM.

We followed to confirm the involvement of FHND004 targeting PBK in the MAPK pathway by detecting the expression of ERK1/2 and p-ERK1/2 in PBK-WT, PBK-OE MM cells with/without the treatment of FHND004. Results showed that PBK overexpression increased the level of p-ERK1/2, while the addition of FHND004 treatment substantially down-regulated the expression of p-ERK1/2, both in ARP1 and H929 cells (Figure 7A, 7B and quantified in Supplementary Figure 2B, 2C). Furthermore, the Co-IP experiment performed that PBK indeed interacted with p-ERK1/2, and the enriched p-ERK1/2 level was reduced after the addition of FHND004 (Figure 7C). In summary, FHND004 could target PBK and inhibit the binding between PBK and p-ERK1/2, thus the decreased phosphorylation of ERK1/2. Hence, our results suggested that FHND004 inhibited MM cell proliferation via the MAPK pathway *in vitro* and *in vivo*. Taken together, the above results suggest that PBK could be a novel drug target for the treatment of MM, and its inhibitor FHND004 will hold promise as an effective agent for anti-MM therapy (Figure 7D).

## DISCUSSION

Despite the continuous improvement in MM treatments, the etiology of MM is not completely clear at present and most patients eventually develop drug resistance and relapse [25–28]. More treatment options for myeloma are needed to be explored. It has been shown that IL-6 (Interleukin-6), as a critical cytokine, can influence the pathogenesis of myeloma through direct activation of PI3K/AKT and MAPK pathways [29, 30]. Moreover, HB-EGF (Heparin-binding EGF-like growth factor) can enhance the survival and the proliferation of IL-6-dependent MM cells. Hence, the EGFR inhibitors with the inhibitory effect on HB-EGF can be used in the combination with IL-6 monoclonal antibody or dexamethasone to achieve better therapeutic effects for MM [31, 32]. The present study inadvertently found that FHND004, derived from the third-generation EGFR-TKI AZD9291 (Osimertinib) which is mainly for solid tumor NSCLC harboring EGFR-sensitive mutations, also has a strong inhibitory effect on the proliferation of MM cells. Since MM cells were assumed to be resistant to EGFR-TKI due to rare EGFR mutations and frequent mutations of KRAS/NRAS/BRAF downstream of EGFR signaling pathway, and previously reported to be moderate or less sensitive to first/second generation TKI (Gefitinib/Afatinib) in a cellular context of NRAS mutation dependent manner [21], we thus hypothesized to find and validate the drug target of FHND compounds in MM cells that would be explored as new therapeutic directions in anti-myeloma therapies.

EGFR-TKIs are extensively developed targeting EGFR, one well-known oncogene altered in many cancers. Our experiments started with the new FHND compounds which were designed and synthesized based on the structure of the marketed third-generation EGFR-TKI AZD9291 (Osimertinib) with an expanded indole ring (FHND004) or a modified pyrimidine ring (FHND008). The antitumor effects of these two compounds are similar in inhibiting MM cell growth, inducing apoptosis (Figure 1D–1I), and inhibiting MAPK signaling pathway (Figure 2), though FHND008 seems to have higher IC_50_ value in both ARP1 and H929 cells (Figure 1C). The molecular docking, and MST results indicated a superior interaction of FHND004 to PBK than that of FHND008 (Figure 4), possibly due to the approaching of the expanded indole ring of FHND004 into the pocket harboring residue D125 of PBK. Recently, the first 4th generation of EGFR-TKI (an allosteric inhibitor EAI045) does not work as a single agent, and can effectively inhibit the kinase activity of T790M/C797S only by combining it with the anti-EGFR mAb Cetuximab [33]. It appears that the focus of tyrosine kinase activity-dependent activity of EGFR may narrow-down and limit the anti-EGFR therapies only to TKI-resistant fractioned patients, attracting more attention to other EGFR kinase-independent roles in cell pro-growth and pro-survival [19]. Mechanistically, in terms of biological significance, the EGFR-TKIs and mAbs exhibit obvious growth inhibition effect rather than inducing DNA fragmentation thus affecting cell survival in many types of EGFR-positive cancer cells, while our FHND004 affects both MM cell growth (increase in cell numbers or size) and survival (ability to keep alive under stresses) by targeting PBK rather than EGFR kinase in MM. Therefore, this minor and critical difference underscores the need to rethink and extend the EGFR kinase-independent directions for more drug targets [19].

PBK, screened and identified as a potential novel drug target in this study, is emerging as promising drug target [34]. Also known as T-lymphokine-activated killer-cell-originated protein kinase (TOPK), PBK is a mitotic serine/threonine protein kinase as a new member of the Mitogen-activated protein kinase kinase (MAPKK) family [35]. It was reported to be involved in a variety of cell processes, such as cell cycle regulation and mitotic progression [36, 37], DNA damage and repair [38] and immune response and inflammation [38–40]. PBK is almost undetectable in normal tissues, except in testis and fetal tissues [41, 42], but is highly expressed in a variety of proliferative malignant cells [43, 44], making it an ideal and effective target with diverse therapeutic potential. However, PBK has been less studied in MM with only a few mentions [45–47] and one genomic data-mining and siRNA screen study [48]. Our study took advantage of transcriptomic RNA-seq, protein chip, molecule docking, MST, and other cellular experiments to characterize the potential drug target by FHND004 *in vitro*. Interestingly, we observed a seemingly more pronounced inhibitory effect as well as p-ERK1/2 in H929 cells than that in ARP1 cells (Figures 1F, 1G, 2D, 2E). It might be at least partially due to the relatively lower background expression level of PBK in H929 compared to ARP1 (Figure 6A), and that H929 cells bearing a confirmed/observed heterozygous G13D mutation in NRAS gene (https://www.keatslab.org/myeloma-cell-lines) may confine and sensitize H929 cell to interact with FHND004. The overexpression intensity in the two MM cells (Figures 6A, 7A, 7B), further supported the interaction between PBK and FHND004. However, whether the inhibition of PBK by binding these two sites, namely N124 and D125, is worth further exploration, which provides a new basis for the development of PBK inhibitors.

In fact, PBK can act on a variety of downstream molecules, such as p38, H3, H2AX, ERK1/2, MKP1, and Prx1 (ref. [37, 40, 49–52]), thus promoting the occurrence of cancer, development, metastasis, and drug resistance [53–56]. PBK promotes autophagy in ovarian cancer cells by phosphorylating ERK1/2 and thereby activating the mTOR pathway while increasing cisplatin resistance [57]. Our results demonstrated that PBK can affect the proliferation of MM by regulating ERK1/2 phosphorylation in the MAPK pathway, and this is an entirely new discovery. Meanwhile, MEK1/2 was considered to be the most suitable target for early tumor suppression, but the prevalent resistance to MEK inhibitors makes MEK1/2 not a suitable target for tumor treatment [58]. However, PBK is considered an oncogenic form of MEK1 and is active in cancer cells while largely unexpressed in normal tissues, which makes PBK more valuable as a promising antitumor target [50, 59]. One of the limitations of this study might be the incomplete characteristics of the involvement of the MAPK pathway from upstream RTKs (including the exact intact/overexpressed EGFR activity) to downstream targets in MM.

In conclusion, PBK is shown to be a novel therapeutic target in MM, and the discovery of possible active sites by FHND004 provides a new approach for MM targeted therapy [32], which may be an alternative choice for the usage of PBK inhibitors.

## MATERIALS AND METHODS

### Cell lines and cell culture

Human MM cell lines ARP1 and H929, and Mouse MM cell line 5TMM3VT cells were cultured in RPMI-1640 (C3010-0500, VivaCell). HEK293 cells were cultured in DMEM (C3110-0500, VivaCell, China). The culture medium contained 10% fetal bovine serum (A6901FBS-500, Invigentech, USA), and 1% penicillin/streptomycin (C100C5, NCM Biotech, China). All cells were incubated at 37° C in a humidified 5% CO2 incubator.

### Antibodies and reagents

All primary antibodies were diluted at a ratio of 1:1000, and all of them were purchased from Cell Signaling Technology (Danvers, MA, USA) except for PBK, which was obtained from Proteintech Group (Wuhan, China), the specific antibody information was as follows: Cleaved Caspase-3 (9661S); PBK (16110-1-AP); PARP (9542S); ERK1/2 (4695S); p-ERK1/2 (4370S); β-actin (4970S). The second antibodies were purchased from Santa Cruz (Santa Cruz, CA, USA) and diluted 1:5000, including goat anti-Rabbit IgG (sc-2005) and rabbit anti-mouse IgG (sc-2004). 3-(4,5-dimethylthiazol-2-yl)-2,5-diphenyltetrazolium bromide assay (MTT) was acquired from Solarbio (Shanghai, China). Puromycin was obtained from Merck KGaA (Darmstadt, Germany). FHND004 and FHND008 were synthesized in Dr. Yongqiang Zhu’s lab at Nanjing Normal University, Nanjing, China.

### Gene expression profiling (GEP)

The gene expression profiling (GEP) of MM patients was collected using the public clinical datasets (GSE2658 and GSE5900). The total therapy 2 (TT2, GSE2658) and the Dutch-Belgian cooperative trial group for hematology-oncology group-65 (HOVON65, GSE19784) trial patient cohort were used in these analyses [45, 60, 61].

### Plasmids and transfection

The plasmids incorporating human PBK cDNA (NM_018492.4) were purchased from Genechem (Shanghai, China). The coding sequence of PBK was cloned into the Flag-tagged lentiviral vector of pTSB. The target expression vector and packaging plasmids (PLP1, PLP2, and VSVG) were co-transfected into HEK293 cells by using liposomal transfection reagent (40802ES02, Yeasen, China), and cultured for 48 h. The supernatant was then collected as the viral solution. The viral solution was transfected into MM cells for culture and screened by puromycin resistance. Finally, the transduction efficiency and whether the cells were successfully constructed were determined by Western Blotting (WB).

### Cell proliferation

Cell viability was determined by MTT assay, and the assay procedure was as follows: first, the desired cells (6 × 10^3^) were seeded into 96-well plates containing 180 μL of cell suspension and 20 μL of different concentrations of the drug per well and incubated for an appropriate period of time. Then each well was incubated for 4 hours with 20 μL of MTT reagent (5 mg/mL). At the end of incubation, centrifuge at 4000 rpm for 15 min at room temperature, discard the supernatant, and add 150 μL of Dimethyl Sulfoxide (DMSO) to each well. Absorbance was measured spectrophotometrically at 570 nm using a microplate reader (Thermo Fisher Scientific, Waltham, MA, USA).

### Apoptosis analysis and cell cycle assays

Annexin V/PI staining assay was used for apoptosis detection of cells. Firstly, the cells collected in good condition were centrifuged at 1200 rpm for 5 min at 4° C, pre-chilled PBS washed twice. After discarding the supernatant, the cell precipitates were collected and resuspended in 100 μL of binding buffer, followed by the addition of 5 μL each of APC Annexin V (640941, Biolegend, USA) and PI (No. C0080, Solarbio, China) were added, and incubated for 15 min at room temperature under light protection. The detection of treated samples by using flow cytometry and Annexin V positive cells were quantitated.

For cell cycle assays, MM cells at the logarithmic growth stage were collected, and centrifuged at 1200 rpm, 4° C for 5 min, and the cell precipitate was washed once with pre-chilled PBS and resuspended by using 250 μL of pre-chilled PBS. The cell suspension was slowly added dropwise to 5 mL of pre-chilled 70% ethanol and allowed to fix overnight at -20° C in the refrigerator. The next day, the fixed cells were centrifuged at 1200 rpm for 5 min at 4° C. the precipitate was washed twice with cold PBS and resuspended with 250 μL of pre-chilled PBS followed by the addition of 5 μL RNase A (200 μg/mL) and the cells were treated on ice for 1 hour. Then, we added 5 μL of PI (No. C0080, Solarbio) into the solution and incubated it for 15 min at room temperature away from light at least. The cell samples were loaded and analyzed by using flow cytometry.

### Western blotting and co-immunoprecipitation (Co-IP)

Collect MM cells and lyse cells with Radioimmunoprecipitation Assay (RIPA) (WB3100, NCM Biotech) buffer with protease inhibitor (K1007, APExBIO, Beijing, China). BCA Protein Quantification Kit (20201ES90, Yeasen) was used to determine the protein concentration. After the separation by SDS-PAGE, total protein (20 μg) was transferred to a PVDF membrane. The membrane was blocked with a blocking solution for 1 hour at room temperature and incubated overnight at 4° C overnight with the primary antibody, the second antibody was incubated with horseradish peroxidase (HRP) coupling at 25° C for 1 hour. Finally, the Super ECL Detection Reagent (36208ES76, Yeasen) was used to develop the blots. Refer to the protocol of Pierce™ Direct Magnetic IP/Co-IP kit (88828, Thermo Fisher Scientific) for detailed procedures of the Co-IP assay.

### Transcriptomic RNA-sequencing (RNA-seq)

ARP1 WT and H929 WT cells were treated with FHND004 and FHND008. Approximately 5 × 10^6^ WT cells and cells treated with the drug were collected, and centrifuged at 4° C, 1200 rpm for 5 min and the supernatant was discarded. The cell precipitates were washed twice with ice-cold PBS, centrifuged at 1200 rpm for 5 min at 4° C, and the supernatant was discarded. Added 1 mL of TRIeasy lysate (10606ES60, Yeasen) to the cell precipitate, followed by mixing well, and then transferred to a 1.5 mL nuclease-free EP tube on dry ice for RNA-seq sequencing. All data analysis and processing are conducted by RealoMics Biotechnological Co., LTD (Shenzhen, China) and deposited in NCBI GEO database (accession number GSE223166).

### Protein microarray technology

The establishment of human proteome chip was provided by Guangzhou Bochong Biotechnology Co., Ltd. (Guangzhou, China). PBK recombinant protein interaction screening based on HuProt™ human whole proteome chip HuProt_V4. The technical route includes: chip closure, co-incubation of biotin-labeled FHND008 and protein chip at room temperature for 1 h, chip cleaning, chip drying, and data reading.

### Microscale thermophoresis assay (MST)

First, PBK protein was fluorescently labeled according to the instructions of the Monolith™ RED-NHS second-generation protein labeling kit. The labeled buffer NHS was used to pass the column, and then the PBK purified protein samples were passed the column as same, collected after centrifugation, the dye obtained by mixing RED-NHS second-generation dye. NHS labeled buffer solution was mixed with PBK purified protein sample for incubation. The incubated dye-PBK protein labeled reactant solution and binding reaction buffer solution were passed through the column to obtain labeled protein samples. Finally, the drug was incubated with PBK protein, and the mixed solution was absorbed by capillary, then the combination ability was tested, and the Kd value was calculated by the MO. Control analysis software.

### 5TMM3VT mouse model

5TMM3VT mouse myeloma cells (1 × 10^6^) were administered intravenously to the 6-week-old C57BL/KaLwRij mice (n = 10 in each group). 2 days later, FHND004 was administered by gavage twice a week at a dose of 20 mg/kg and would be sacrificed until they exhibited signs of hind limb paralysis. Record the survival time of each group of mice and plot the survival curves.

### Patient-derived tumor xenograft (PDX) model

The patient-derived tumor xenograft (PDX) model was created by using a biopsy sample of a cutaneous subcutaneous extramedullary tumor on the head skin of a MM patient collected from the Department of Hematology, the First Affiliated Hospital of Nanjing Medical University. Under pentobarbital anesthesia, the 2~3 small blocks of 2*2*2 mm^3^ extramedullary biopsy tumor sections were subcutaneously transplanted into 4 to 6-week-old male SCID/NOD mice without any sublethal irradiation required (n = 12). When the tumors reached a size of 500 mm^3^, they were harvested and the tumor tissues were then divided into small pieces and implanted under the skin again. This operation was repeated three times until the tumor size grew to 100~150 mm^3^, the mice were assigned randomly into control and treatment groups and PBS and FHND004 were injected respectively. The dosing frequency was twice a week [62].

### Statistical analyses

All values were expressed as mean ± SD unless otherwise specified. Statistical differences were analyzed in two separate ways, using the Student’s t-test for two independent experimental groups and the one-way ANOVA for three or more experimental groups. The threshold p-values were set at *p* < 0.05 (*), *p* < 0.01 (* *) and *p* < 0.001 (* * *). Statistical analyses were performed using SPSS version 19.0 and GraphPad Prism 8.0 software.

## Supplementary Material

Supplementary Figures

Supplementary Tables
