# Supplementary material for: FHND004 inhibits malignant proliferation of multiple myeloma by targeting PDZ-binding kinase in MAPK pathway
Source: Aging (Albany NY). 2024 Mar 7;16(5):4811–31. doi: 10.18632/aging.205634 (PMC10968680; doi:10.18632/aging.205634)
Supplement: Supplementary Figures [file aging-16-205634-s001.pdf]

SUPPLEMENTARY FIGURES

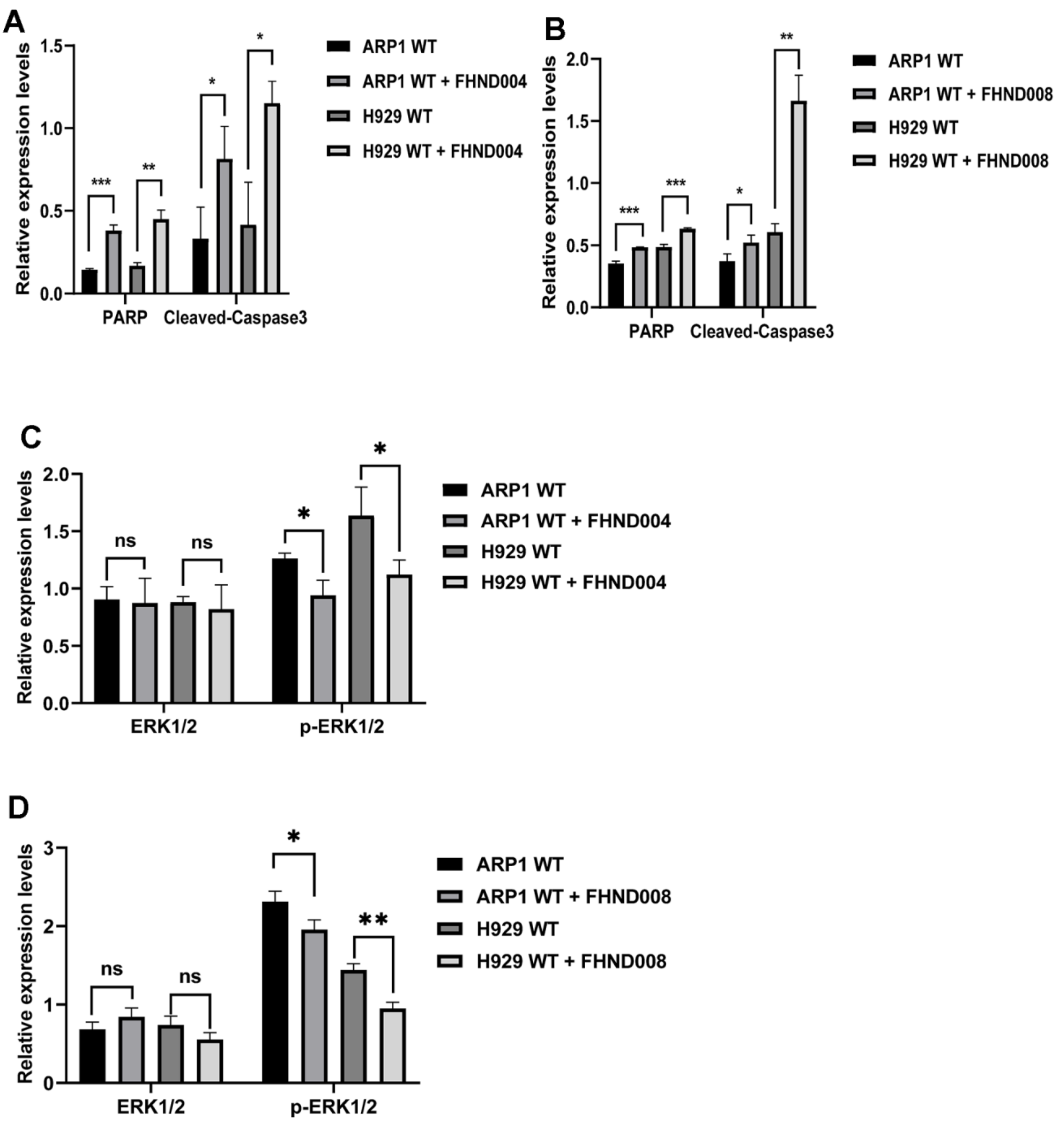

Supplementary Figure 1. Quantitative analysis of WB results. (**A**, **B**) Relative expression of PARP and Cleaved Caspase-3 in ARP1 WT, H929 WT with or without treatment of FHND004 (4 μM) (**A**) and FHND008 (4 μM) (**B**). (**C**, **D**) Relative expression of ERK, p-ERK in ARP1 WT and H929 WT cells with or without treatment of FHND004 (4 μM) (**C**) and FHND008 (4 μM) (**D**). The data are expressed as mean ± SD. (*$p < 0.05$; ** $p < 0.01$; ***$p < 0.001$). Related to Figures 1F, 1G, 2D, 2E in the manuscript.

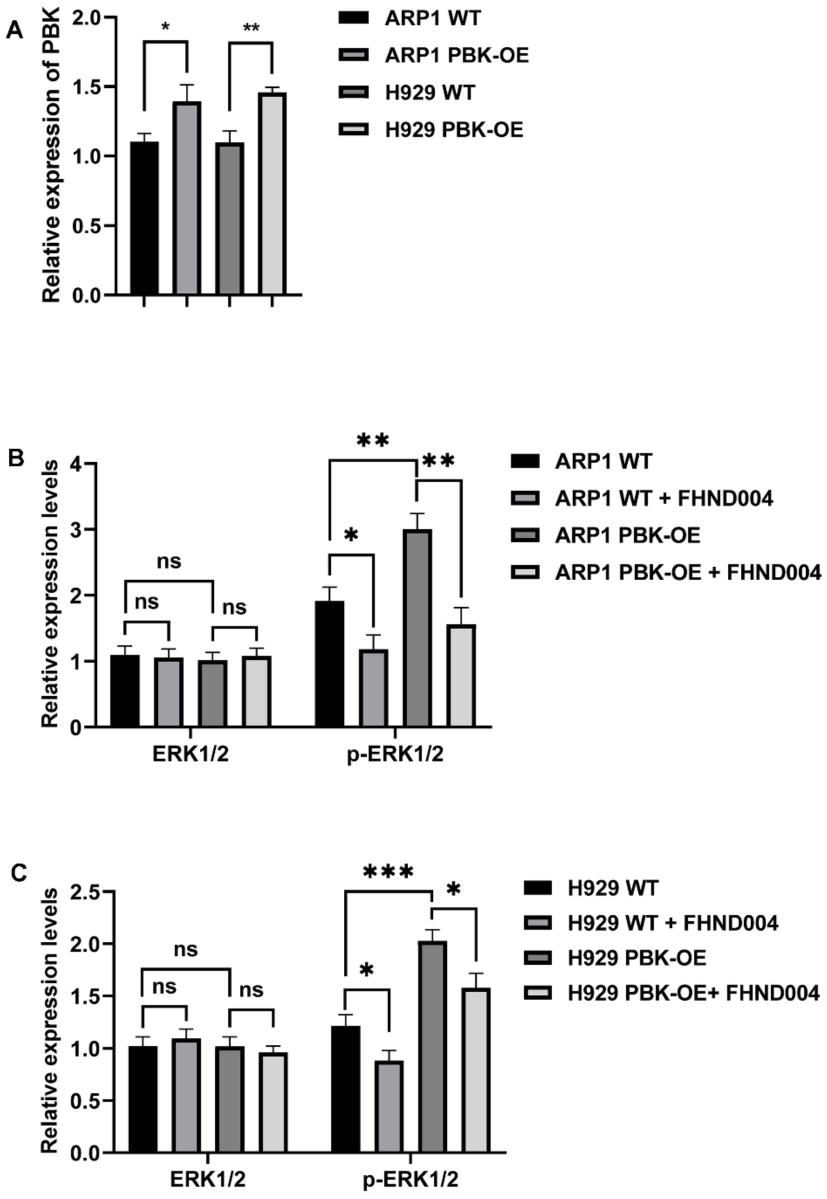

Supplementary Figure 2. Quantitative analysis of WB results. (**A**) Relative expression of PBK in ARP1 and H929 WT and PBK-OE cells. (**B**, **C**) Relative expression of ERK, p-ERK in PBK-WT and PBK-OE cells with or without treatment of FHND004 (4 μM) (**A**: ARP1, **B**: H929). The data are expressed as mean ± SD. (*$p < 0.05$; ** $p < 0.01$; ***$p < 0.001$). Related to Figures 6A, 7A, 7B in the manuscript.
