# Supplementary material for: FHND004 inhibits malignant proliferation of multiple myeloma by targeting PDZ-binding kinase in MAPK pathway
Source: Aging (Albany NY). 2024 Mar 7;16(5):4811–31. doi: 10.18632/aging.205634 (PMC10968680; doi:10.18632/aging.205634)
Supplement: Supplementary Tables [file aging-16-205634-s002.pdf]

SUPPLEMENTARY TABLES

Supplementary Table 1. The effects of FHND drugs on cell viability of MM cells.

FHND drugs	ARP1 WT	H929 WT
FHND002 (μM)	7.98	15.84
FHND004 (μM)	3.95	2.84
FHND006 (μM)	10.77	/
FHND007 (μM)	/	40.64
FHND008 (μM)	5.68	/
FHND009 (μM)	16.52	18.59

Supplementary Table 2. Potential proteins interacting with FHND008 based on HuProt™ human proteomic chip V4.0.

Block	Column	Row	Name	ID	SNR-biotin	SNR-FHND008	Normalization-biotin	Normalization-FHND008-biotin	Fold change
9	15	57	MYLK	JHU14956.B9C15R57	1.86833333333333	3.52971428571429	1.86833333333333	6.06282849990836	3.24504647631134
1	25	19	GDPD5	JHU01374.B1C25R19	2.51282051282051	3.49350259896042	2.51282051282051	6.00062934476153	2.38800555556836
1	29	47	GADD45G	JHU02812.B1C29R47	2.29787234042553	2.62281976744186	2.29787234042553	4.50509733904754	1.96055161977069
14	23	73	CYAT1	JHU15234.B14C23R73	2.80876068376068	2.47417582417582	2.80876068376068	4.24977844844512	1.51304398164497
11	23	85	TCEB2	JHU03155.B11C23R85	2.17142857142857	2.33883333333333	2.17142857142857	4.0173068532088	1.85007552450405
3	25	25	MARC2	JHU01787.B3C25R25	1.96332046332046	2.30470822281167	1.96332046332046	3.95869171445079	2.01632478671142
8	27	51	PPM1G	JHU08990.B8C27R51	2.47794117647059	2.1789825282631	2.47794117647059	3.74273844957469	1.51042263840403
11	9	27	DOHH	JHU13137.B11C9R27	2.28323170731707	2.05241935483871	2.28323170731707	3.52534668560619	1.54401617422731
7	8	47	ACAA1	JHU08544.B7C8R47	2.20482261640798	1.9347024256064	2.20482261640798	3.32314970995865	1.50721862395107
2	3	87	SNX15	JHU05453.B2C3R87	1.87	1.92758291577189	1.87	3.31092085412611	1.77054591129739
6	8	27	PBK	JHU07458.B6C8R27	1.75295508274232	1.824	1.75295508274232	3.13300122579043	1.78726839987775
13	1	75	FABP5	JHU00992.B13C1R75	1.57619047619048	1.79357394366197	1.57619047619048	3.08073978291597	1.95454789852675
16	6	53	CCDC117	JHU18658.B16C6R53	1.96150362318841	1.79356805664831	1.96150362318841	3.08072967106234	1.57059596252728
5	3	33	IGLC2	JHU07717.B5C3R33	1.5155255395125	1.77923976608187	1.5155255395125	3.05611862286709	2.01654049581386

SNR, Signal-to-noise Ratio.
